# Supplementary material for: Discovery of neutralizing SARS-CoV-2 antibodies enriched in a unique antigen specific B cell cluster
Source: PLoS One. 2023 Sep 20;18(9):e0291131. doi: 10.1371/journal.pone.0291131 (PMC10511142; doi:10.1371/journal.pone.0291131)
Supplement: S2 Fig — (PDF) [file pone.0291131.s002.pdf]

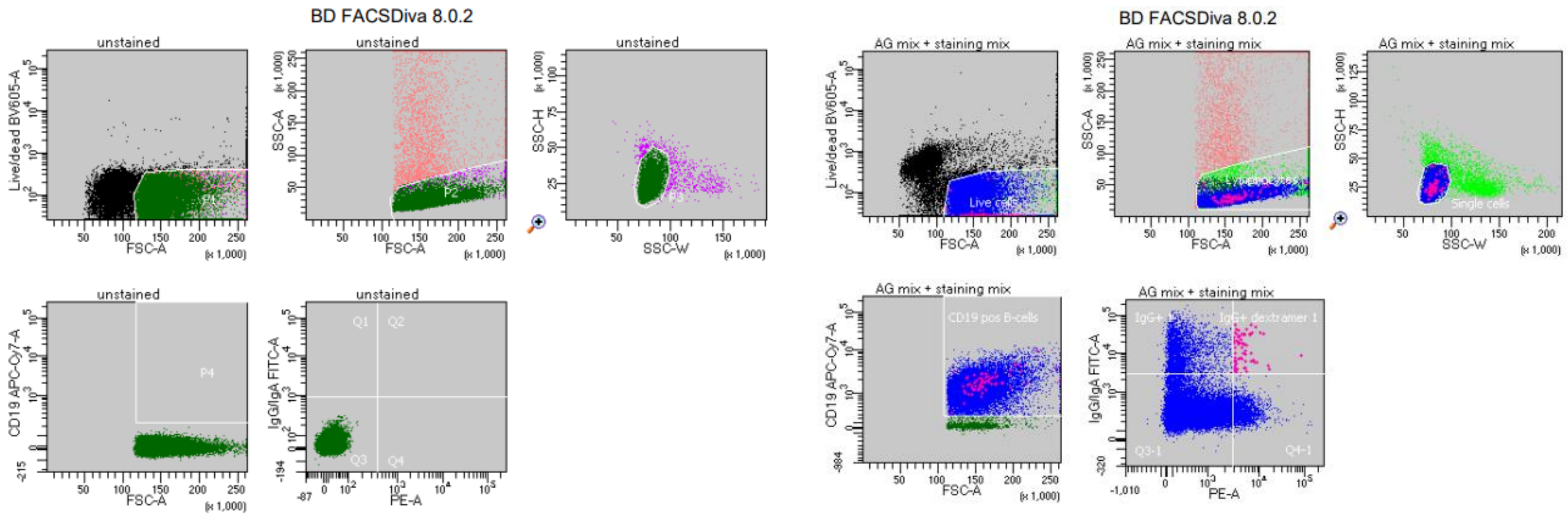

Experiment Name: Sym030 patient material_001
Specimen Name: patient #mix Covid-019_Group 3
Tube Name: unstained
Record Date: Dec 9, 2020 10:16:04 AM

Population	#Events	%Parent	%Total
All Events	50,000	####	100.00
P1	34,771	69.54	69.54
P2	31,138	89.55	62.28
P3	30,436	97.75	60.87
P4	0	0.00	0.00
Q1	0	####	0.00
Q2	0	####	0.00
Q3	0	####	0.00
Q4	0	####	0.00

Experiment Name: Sym030 patient material_001
Specimen Name: patient #mix Covid-019_Group 3
Tube Name: AG mix + staining mix
Record Date: Dec 9, 2020 12:49:18 PM

Population	#Events	%Parent	%Total
All Events	999,811	####	100.00
Single cells	647,918	92.73	64.80
CD19 pos B-cells	624,698	96.42	62.48
IgG+ 1	59,328	9.50	5.93
IgG+ dextramer 1	1,232	0.20	0.12
Q3-1	523,043	83.73	52.31
Q4-1	41,095	6.58	4.11
Live cells	764,678	76.48	76.48
Lymphocytes	698,695	91.37	69.88

S2 Figure: FACS gating strategy

Sorting procedure for sorting antigen specific memory B-cells. **Left**) Enriched B cells were tested in sort setup with unstained cells to set borders for the positive and negative cells. **Right**) Example of plots, with live gate, singlet gate, antigen specific IgG1/IgA expressing B-cells are shown back-gated in magenta. For the sort shown here, 0.12% of the total number of cells in the sample were antigen specific switched memory B-cells.
