# Supplementary material for: Discovery of neutralizing SARS-CoV-2 antibodies enriched in a unique antigen specific B cell cluster
Source: PLoS One. 2023 Sep 20;18(9):e0291131. doi: 10.1371/journal.pone.0291131 (PMC10511142; doi:10.1371/journal.pone.0291131)
Supplement: S3 Fig — (PDF) [file pone.0291131.s003.pdf]

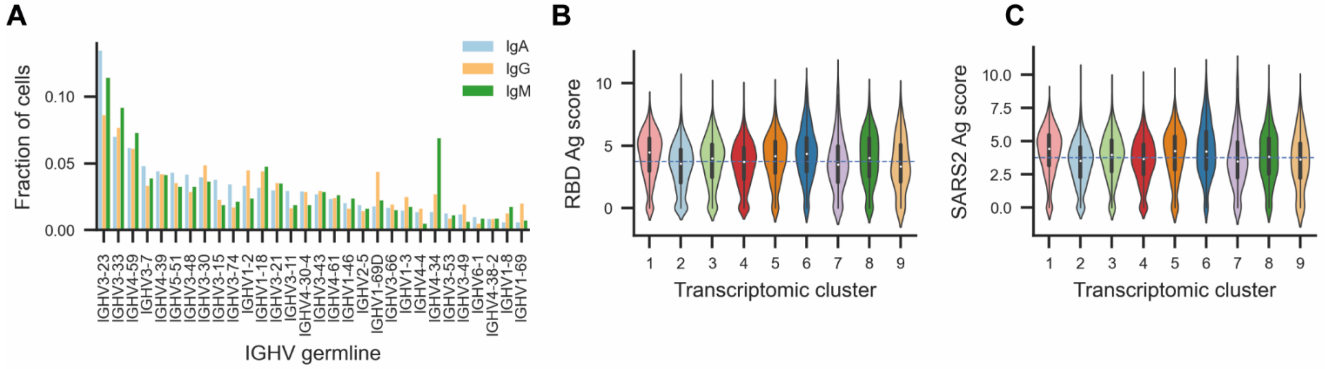

D

Selection	IgA1	IgG1	IgA2	Total
Cluster 6	75	87	11	173
All memory B cells	116	53	5	174
Cluster 8	56	48	4	108
Total	247	188	20	455

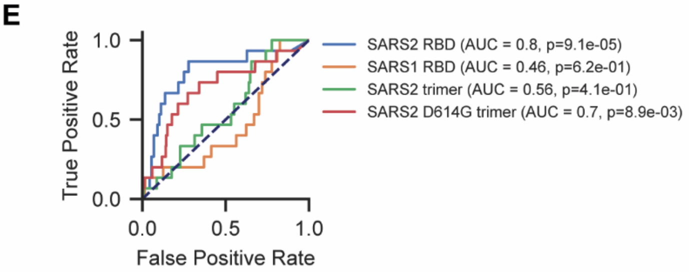

S3 Figure: Characteristics of the single-cell population.

A Fraction of B cells utilizing IGHV subsets for the 30 most frequently used genes, stratified by isotype. Significant differences are observed between IgG and IgM cells for *IGHV4-34*, between IgG and IgA for *IGHV3-23*, *IGHV3-15*, *IGHV3-74*, *IGHV3-11*, *IGHV1-69D*, *IGHV4-34*, *IGHV1-3*, and *IGHV1-69*, and between IgA and IgM for *IGHV4-34* and *IGHV1-8* (false discovery rate <0.05, two-proportions Z test).

B Ag score distribution for RBD stratified by transcriptomic cluster with the mean Ag score given as a dotted line.

C. Ag score distribution for the trimeric SARS2 spike protein stratified by transcriptomic cluster.

D Number of mAbs selected from the population.

E Predictive performance of the Ag scores against binding to the RBD domain of SARS2 spike protein. The SARS-CoV-1 (Orange) Ag score and the SARS-CoV-2 trimer Wuhan strain (Green) did not show significant correlation with binding. SARS-CoV-2 D614G Trimer (Red).
