# Supplementary material for: Discovery of neutralizing SARS-CoV-2 antibodies enriched in a unique antigen specific B cell cluster
Source: PLoS One. 2023 Sep 20;18(9):e0291131. doi: 10.1371/journal.pone.0291131 (PMC10511142; doi:10.1371/journal.pone.0291131)
Supplement: S4 Fig — (PDF) [file pone.0291131.s004.pdf]

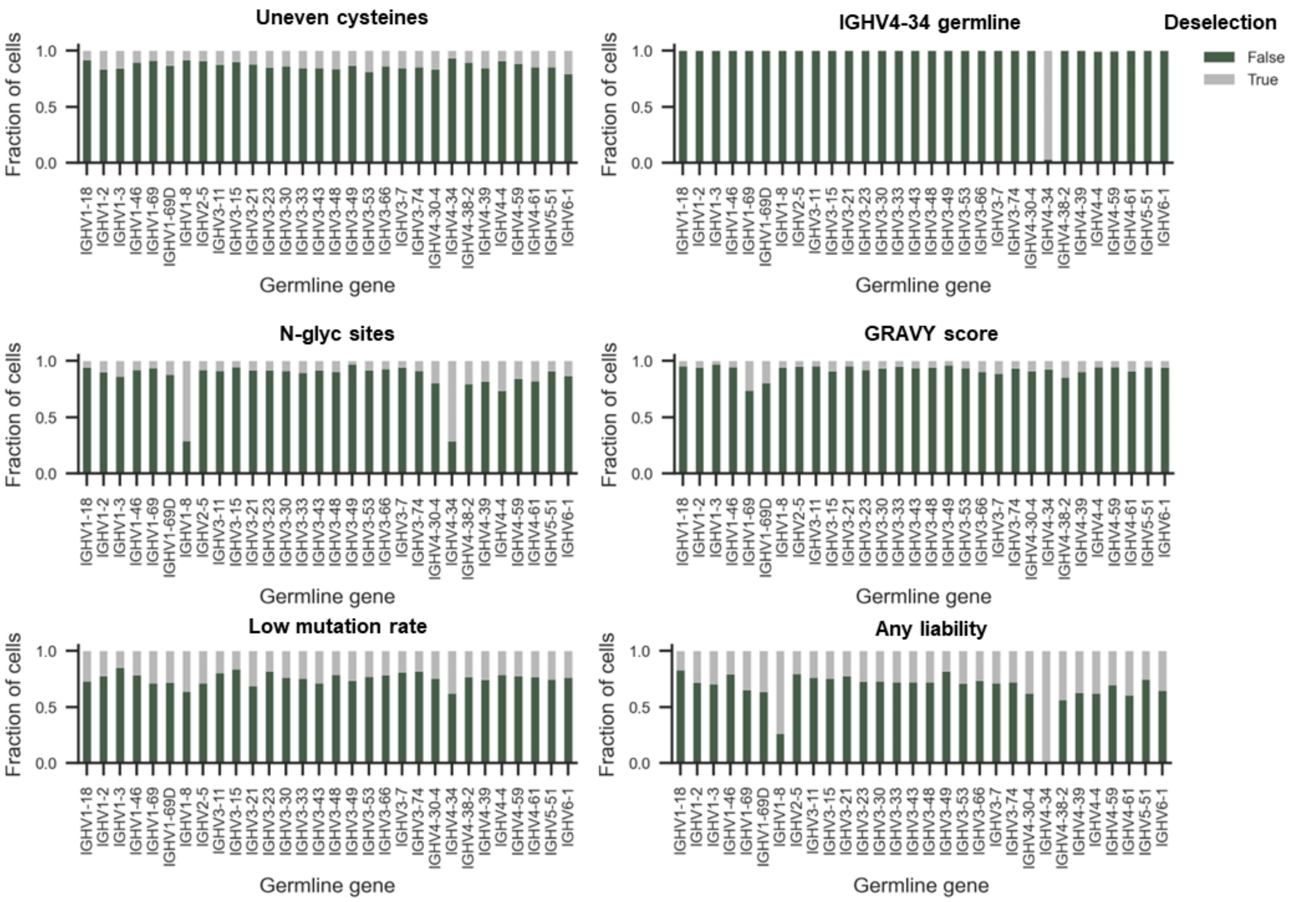

S4 Figure: Drug development liabilities

The five drug development liabilities selection criteria and how they impacted germline sequences selection possibilities.
