# Supplementary material for: Discovery of neutralizing SARS-CoV-2 antibodies enriched in a unique antigen specific B cell cluster
Source: PLoS One. 2023 Sep 20;18(9):e0291131. doi: 10.1371/journal.pone.0291131 (PMC10511142; doi:10.1371/journal.pone.0291131)
Supplement: S5 Fig — (PDF) [file pone.0291131.s005.pdf]

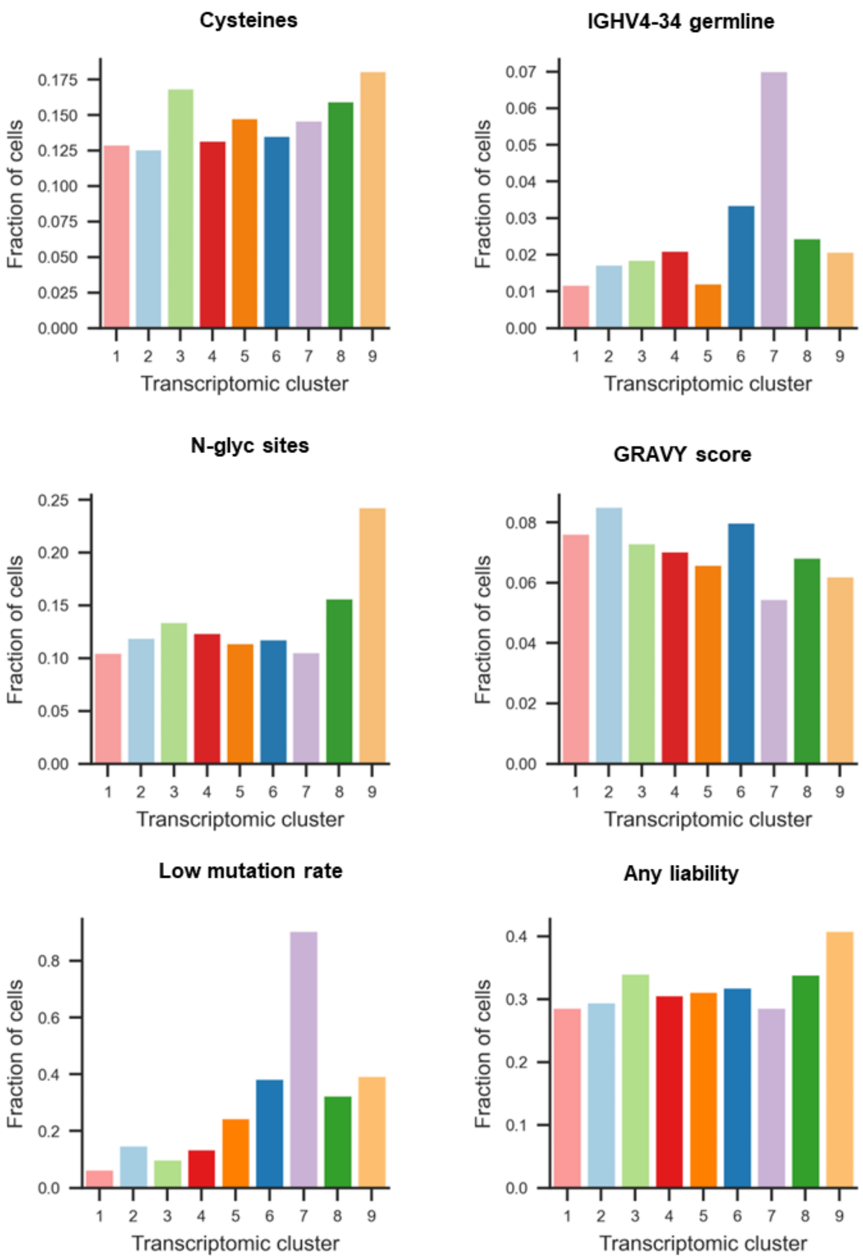


**S5 Figure: Cluster distribution of drug development liabilities.**

The five drug development liabilities selection criteria and how they impacted the UMAP cell cluster selection possibilities.
