# Supplementary material for: Discovery of neutralizing SARS-CoV-2 antibodies enriched in a unique antigen specific B cell cluster
Source: PLoS One. 2023 Sep 20;18(9):e0291131. doi: 10.1371/journal.pone.0291131 (PMC10511142; doi:10.1371/journal.pone.0291131)
Supplement: S6 Fig — (PDF) [file pone.0291131.s006.pdf]

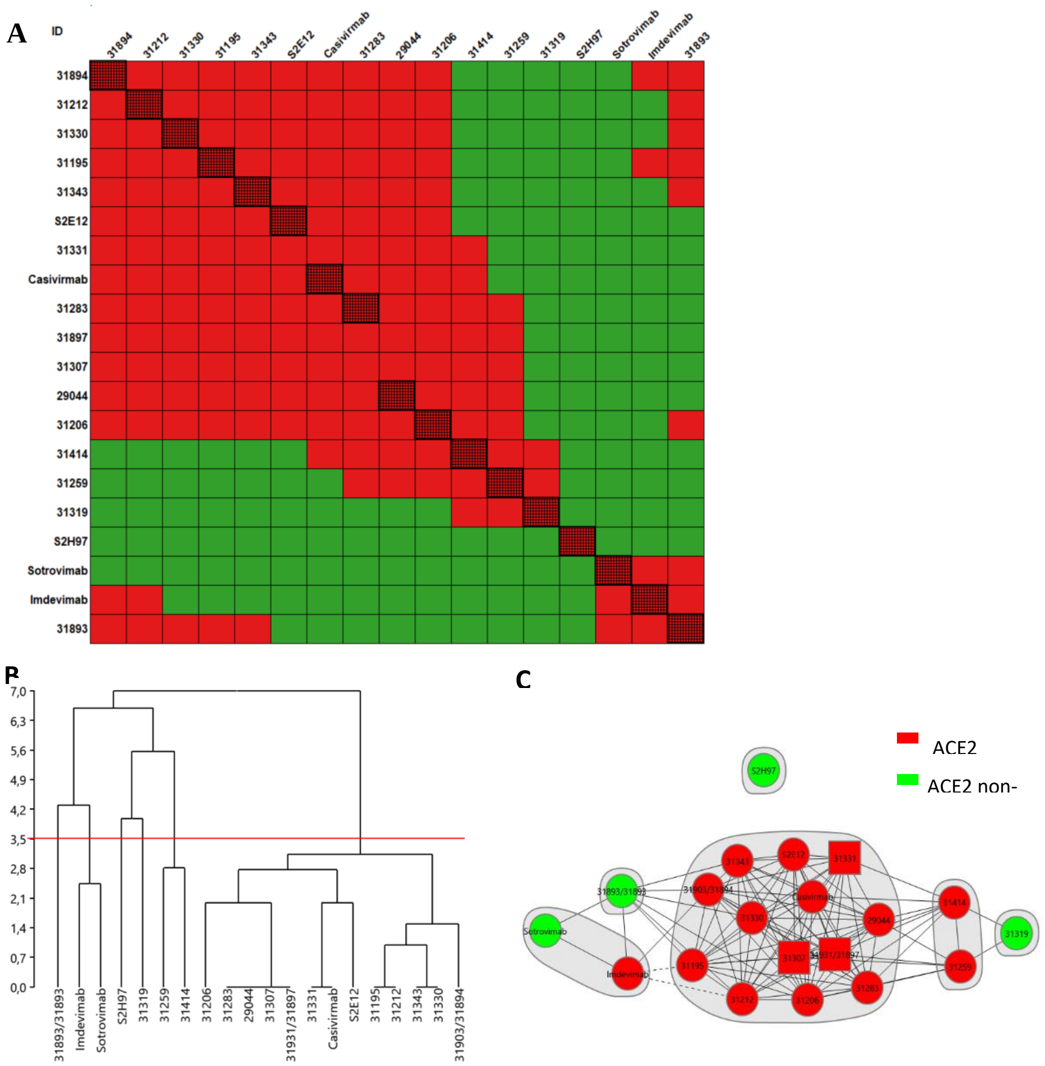

S7 Figure: Surface Plasmon Resonance competition data

A Heat map of epitope binning analysis of mAbs binding SARS-CoV-2 RBD. Red boxes indicate mAbs competing for the same antigen binding site. Diagonal black boxes, show self-self mAb competition. Green boxes are sandwiching mAbs, where immobilized mAbs and mAb in solution can bind the antigen simultaneously.

B Dendrogram showing the hierarchically clustering of mAbs based on their competition profiles. The red line shows a cut-off of 3.5 to define epitope community assignment.

C Epitope community plot overlayed with ACE2 competitions at 50 nM. Red color indicates mAbs blocking ACE2 binding and green color indicate mAbs that bind the SARS-CoV-2 RBD simultaneously with ACE2 receptor binding. Lines connect competitive mAbs, and grey envelopes highlight epitope communities as defined from the dendrogram in b.
