# Supplementary material for: Discovery of neutralizing SARS-CoV-2 antibodies enriched in a unique antigen specific B cell cluster
Source: PLoS One. 2023 Sep 20;18(9):e0291131. doi: 10.1371/journal.pone.0291131 (PMC10511142; doi:10.1371/journal.pone.0291131)
Supplement: S7 Fig — (PDF) [file pone.0291131.s007.pdf]

A

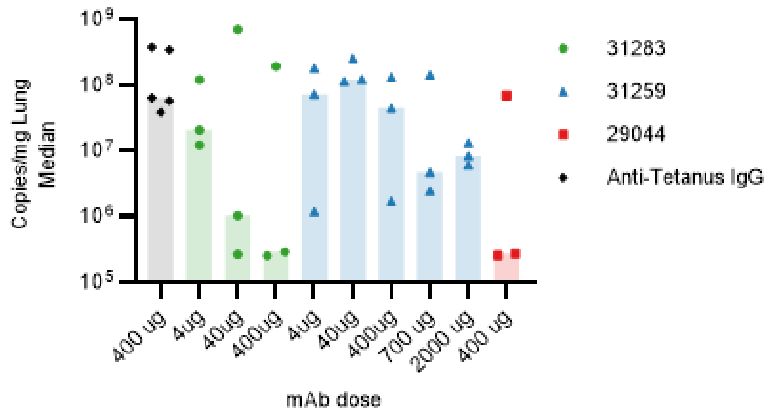

B

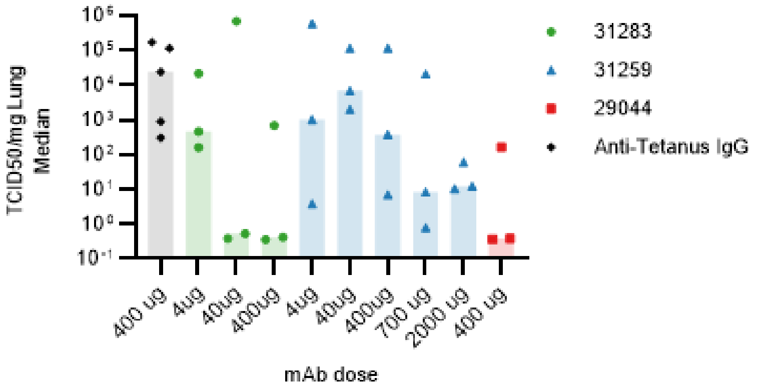

S8 Figure: SARS-CoV-2 lung viral loads

A Copies of SARS-CoV-2 viral RNA/mg mouse lung analyzed by ddPCR. Data shows single treatment dose of either 31283, 31259 or 29044 given 24 hours prior to SARS-CoV-2 nasal exposure. Bars marks the median for all treatment groups, and symbols represent all individual mice.

B Tissue Culture Infections Dose of SARS-CoV-2 per mg mouse lung. Data shows single treatment dose of either 31283, 31259 or 29044 given 24 hours prior to SARS-CoV-2 nasal exposure. Bars marks the median for all treatment groups, and symbols represent all individual mice.
