# Supplementary material for: Discovery of neutralizing SARS-CoV-2 antibodies enriched in a unique antigen specific B cell cluster
Source: PLoS One. 2023 Sep 20;18(9):e0291131. doi: 10.1371/journal.pone.0291131 (PMC10511142; doi:10.1371/journal.pone.0291131)
Supplement: S8 Fig — (PDF) [file pone.0291131.s008.pdf]

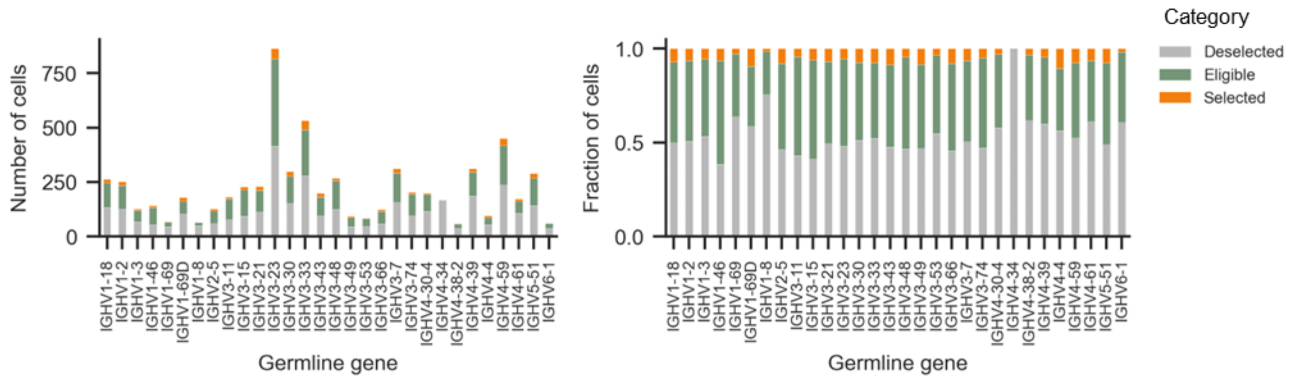

S9 Figure: Impact of germline on selection/deselection.

Left) Total number of cells split by germline sequence and whether the antibody sequence was deselected, Eligible or Selected for monoclonal antibody production and testing. **Right**) Similar analysis but normalized to number of cells with a given germline sequence.
