# Supplementary material for: Discovery of neutralizing SARS-CoV-2 antibodies enriched in a unique antigen specific B cell cluster
Source: PLoS One. 2023 Sep 20;18(9):e0291131. doi: 10.1371/journal.pone.0291131 (PMC10511142; doi:10.1371/journal.pone.0291131)
Supplement: S9 Fig — (PDF) [file pone.0291131.s009.pdf]

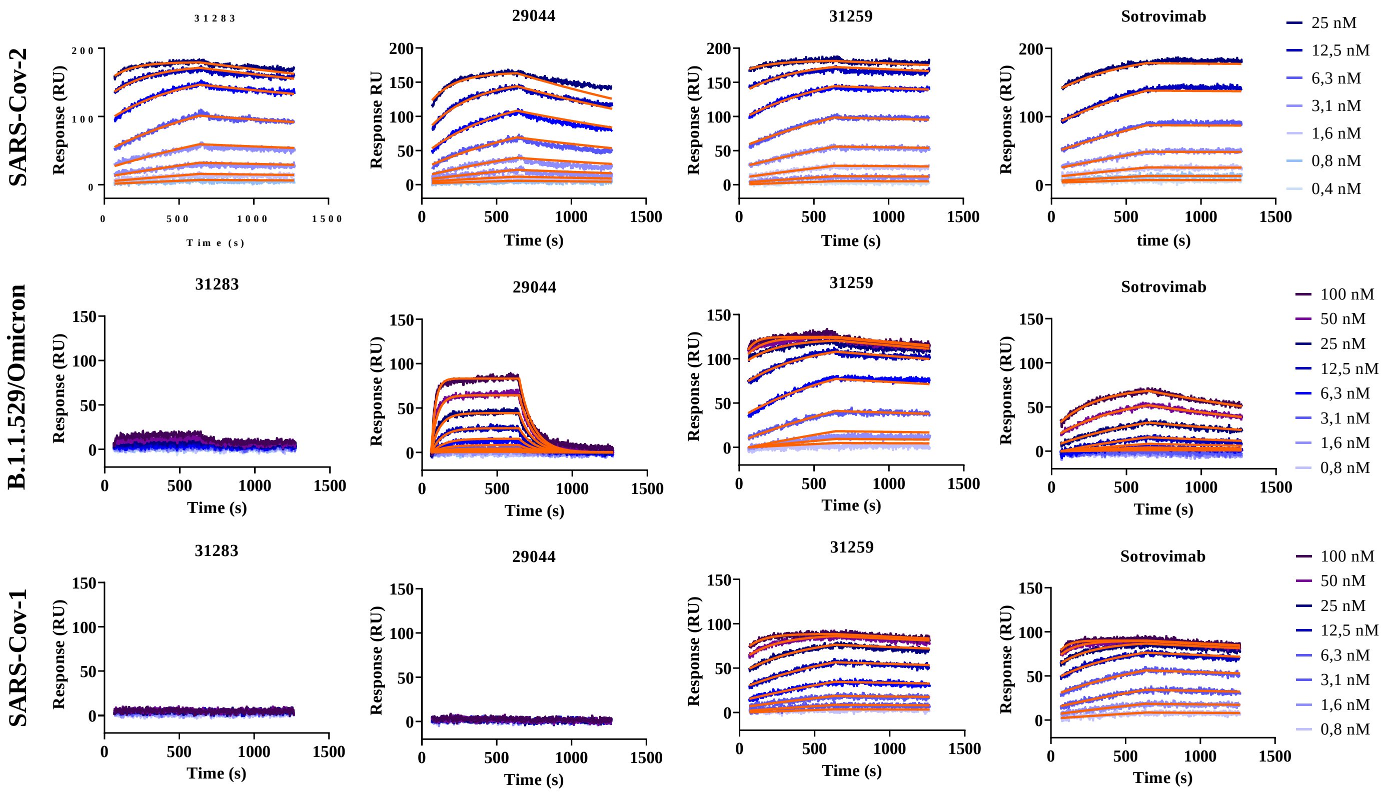

S10 Figure: Surface Plasmon Resonance sensorgrams of RBD variants.

Wild type (Wuhan) SARS-Cov-2 (upper row), B.1.1.529/Omicron (middle row), and SARS-Cov-1 (bottom row) interacting with the mAbs 31283, 29044, 31259 and Sotrovimab. The concentrations of injected RBD are shown to the right for each antigen presented in a row. Data were globally fitted to 1:1 Langumir binding model (orange lines). Kinetic values are shown in supplementary table 2.
