# Supplementary material for: Discovery of neutralizing SARS-CoV-2 antibodies enriched in a unique antigen specific B cell cluster
Source: PLoS One. 2023 Sep 20;18(9):e0291131. doi: 10.1371/journal.pone.0291131 (PMC10511142; doi:10.1371/journal.pone.0291131)
Supplement: S10 Fig — (PDF) [file pone.0291131.s010.pdf]

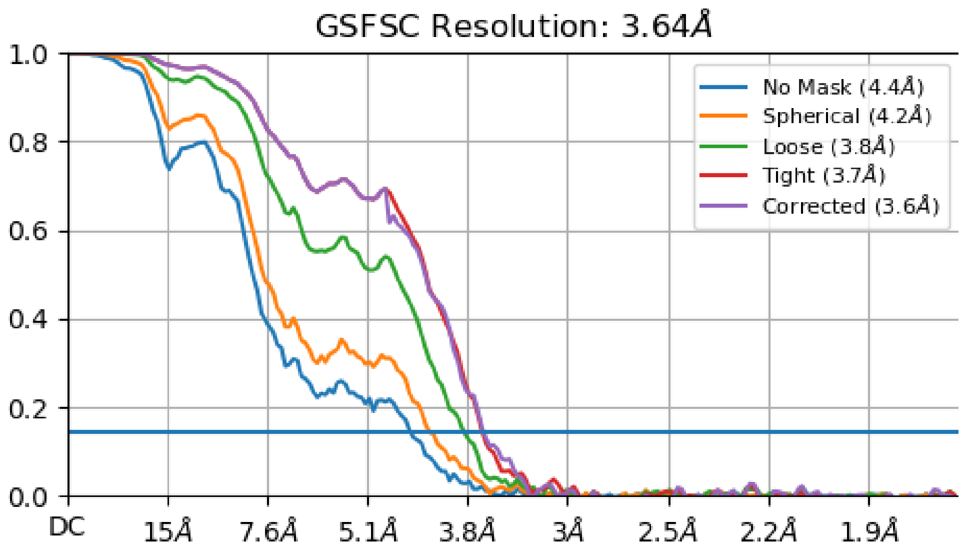

S11 Figure: Cryo-EM refinement

$FSC_{0.143}$ plots for non-uniform refinement of cryo-electron microscopy maps.
