# Supplementary material for: Discovery of neutralizing SARS-CoV-2 antibodies enriched in a unique antigen specific B cell cluster
Source: PLoS One. 2023 Sep 20;18(9):e0291131. doi: 10.1371/journal.pone.0291131 (PMC10511142; doi:10.1371/journal.pone.0291131)
Supplement: S1 Table — (PDF) [file pone.0291131.s011.pdf]

mAb ID	Trimer SARS-CoV-2	RBD SARS-Cov-2	RBD N501Y (Alpha + Beta)	RBD L452R, E484Q (Delta)	RBD K417N, L452R, T478K (Delta variant)	RBD B.1.1.529/Omicron	RBD SARS-Cov-1
**31894**	3,1E-11	3,1E-10	1,6E-08		1,1E-07		
**31893**	6,0E-11	1,4E-10	3,7E-09	1,1E-08	1,1E-09	2,9E-07	
**31932**	3,1E-09						
**31307**	4,2E-10	5,8E-10		6,5E-10	1,7E-05		
**31330**	3,2E-11	4,7E-11			3,1E-09		
**31897**	6,4E-10	2,2E-10	1,1E-06	1,5E-10	8,5E-08		
**31283**	2,9E-10	2,2E-10	3,4E-09	9,1E-10	3,5E-09		
**31195**	6,9E-11	6,1E-10			6,8E-09		
**31331**	1,9E-10	3,4E-10	6,8E-09	9,7E-10	6,6E-09		
**29044**	7,6E-10	2,0E-09	1,3E-09	5,7E-09	3,1E-08	3,2E-08	
**31282**							
**31319**	3,9E-09	2,6E-10	4,1E-10	2,9E-10	3,2E-10	1,8E-09	7,3E-09
**31259**	3,0E-09	4,4E-11	3,6E-10	5,9E-11	9,3E-11	5,6E-10	9,1E-10
**31206**	3,1E-10	1,3E-09		8,8E-09	2,6E-08		
**31343**	4,0E-11	5,6E-10		3,9E-06	2,1E-08		
**31426**							
**31367**							
**31414**	2,2E-10	4,6E-11	1,2E-10	1,9E-11	5,0E-12	5,7E-10	1,7E-09
**31212**	2,9E-09	2,8E-08			3,9E-08		

1E-12
1E-11
1E-10
1E-09
1E-08
1E-07
1E-06
1E-05
n.b

S6 Figure: Surface Plasmon Resonance data

Affinity (K_D, M) of lead mAbs towards Spike RBD variants measured by SPR. K_D values are mean of 1-8 replicates. n.b = no binding. Colors indicate 10-fold value differences.
