# Supplementary material for: Discovery of neutralizing SARS-CoV-2 antibodies enriched in a unique antigen specific B cell cluster
Source: PLoS One. 2023 Sep 20;18(9):e0291131. doi: 10.1371/journal.pone.0291131 (PMC10511142; doi:10.1371/journal.pone.0291131)
Supplement: S2 Table — (PDF) [file pone.0291131.s012.pdf]

ST1

mAb ID	Neutralization status	Transcriptomic cluster	SHM % (VH/VL avg)	SHM % (VH)	SHM % (VL)	IGHV gene	Isotype VH (IGH gene)	Isotype VL (IGK/IGL gene)	SARS2 D614G Ag score	SARS2 RBD Ag score	SARS2 Ag score	SARS1 RBD Ag score
31184	Top neutralizer	5	2,9	4,6	1,2	IGHV3-23	IGHA1	IGK	5,3	5,6	4,0	2,7
31195	Top neutralizer	8	2,4	2,4	2,4	IGHV1-2	IGHG1	IGK	7,1	7,2	3,2	1,6
31206	Top neutralizer	1	2,0	2,9	1,2	IGHV4-39	IGHG1	IGK	5,0	4,9	1,9	1,9
31243	Top neutralizer	2	5,3	6,8	3,9	IGHV3-23	IGHA1	IGK	6,4	4,5	2,9	1,6
31259	Top + *In-Vivo*	8	2,4	3,3	1,5	IGHV4-4	IGHG1	IGK	2,3	5,1	2,0	2,9
31270	Top neutralizer	8	5,2	7,4	2,9	IGHV3-23	IGHA1	IGK	4,6	4,9	4,1	3,8
31282	Top neutralizer	8	2,2	2,4	2,1	IGHV1-24	IGHA1	IGK	5,8	5,8	4,2	5,1
31283	Top + *In-Vivo*	8	2,4	3,0	1,8	IGHV3-66	IGHG1	IGK	5,8	6,3	2,4	1,3
31295	Top neutralizer	8	6,1	9,2	2,9	IGHV1-58	IGHG1	IGK	4,6	3,3	1,4	3,0
31307	Top neutralizer	8	2,7	2,7	2,6	IGHV3-66	IGHG1	IGK	5,3	5,9	3,9	2,1
31317	Top neutralizer	8	4,8	4,0	5,6	IGHV3-13	IGHA2	IGK	4,0	2,9	1,7	2,9
31318	Top neutralizer	8	2,8	3,8	1,7	IGHV3-64D	IGHG1	IGK	2,1	3,8	2,1	1,4
31329	Top neutralizer	4	9,8	11,7	7,9	IGHV4-59	IGHA1	IGK	5,3	5,2	3,3	5,7
31330	Top neutralizer	8	2,9	4,6	1,2	IGHV1-69D	IGHG1	IGK	5,9	7,1	1,0	2,3
31331	Top neutralizer	8	2,1	1,6	2,6	IGHV3-66	IGHG1	IGK	6,1	6,5	3,5	1,8
31343	Top neutralizer	8	2,1	1,4	2,9	IGHV3-11	IGHG1	IGK	5,8	6,9	1,4	2,0
31366	Top neutralizer	8	6,8	7,0	6,6	IGHV1-2	IGHA1	IGL	4,9	5,8	5,0	5,2
31367	Top neutralizer	8	5,8	6,0	5,7	IGHV3-33	IGHG1	IGL	8,2	8,5	7,0	7,1
31414	Top neutralizer	8	2,3	1,5	3,1	IGHV5-51	IGHG1	IGL	2,9	7,1	1,9	3,8
31426	Top neutralizer	8	3,4	4,2	2,6	IGHV3-21	IGHA1	IGL	7,1	7,2	5,6	6,3
31893	Top neutralizer	8	2,1	2,1	2,1	IGHV4-59	IGHG1	IGK	4,1	6,3	2,4	1,4
31894	Top neutralizer	9	6,3	9,9	2,6	IGHV1-69D	IGHG1	IGK	4,6	3,0	3,9	2,5
31897	Top neutralizer	3	4,0	5,7	2,4	IGHV1-18	IGHG1	IGK	0,0	0,0	1,8	3,7
31932	Top neutralizer	8	7,4	9,8	5,0	IGHV5-51	IGHA1	IGK	1,6	2,1	2,6	0,0
29044	Top + *In-Vivo*	N/A	2,9	4,5	1,2	IGHV3-66	IGHG1	IGK	N/A	N/A	N/A	N/A

S12 Table: Data on top neutralizing monoclonal antibodies

Table listing all features of the top neutralizing monoclonal antibodies evaluated. SHM: Somatic hypermutation in variable light (VL) or variable heavy (VH), the germline usage and the antigen score from the single cell transcriptomic antigen sequencing.
