# Supplementary material for: Discovery of neutralizing SARS-CoV-2 antibodies enriched in a unique antigen specific B cell cluster
Source: PLoS One. 2023 Sep 20;18(9):e0291131. doi: 10.1371/journal.pone.0291131 (PMC10511142; doi:10.1371/journal.pone.0291131)
Supplement: S3 Table — (PDF) [file pone.0291131.s013.pdf]

ST2

mAb ID	Antigen	n	k_{on} (M-1 s-1)	± SD, k_{on} (M-1 s-1)	k_{off} (s-1)	± SD, k_{off} (s-1)	K_D (M)	± SD, K_D (M)
29044	**Trimer SARS-CoV-2***	8	4,5E+4 ±	3,6E+03	3,4E-05	1,3E-05	7,6E-10	2,9E-10
	**RBD SARS-Cov-2**	8	2,4E+05	1,5E+04	4,8E-04	9,0E-05	2,0E-09	4,0E-10
	**RBD N501Y (Alpha + Beta)**	8	3,3E+05	2,1E+04	4,2E-04	5,3E-05	1,3E-09	1,8E-10
	**RBD L452R, E484Q (Delta)**	6	1,9E+05	7,6E+03	1,1E-03	2,7E-04	5,7E-09	1,4E-09
	**RBD K417N, L452R, T478K (Delta variant)**	8	4,8E+04	1,5E+04	1,2E-03	2,4E-04	3,1E-08	1,2E-08
	**RBD B.1.1.529/Omicron**	8	2,3E+05	1,6E+04	7,1E-03	1,9E-03	3,2E-08	8,8E-09
	**RBD SARS-Cov-1**	8	n.b.	n.b.	n.b.	n.b.	n.b.	n.b.
31259	**Trimer SARS-CoV-2***	8	1,7E+04	1,6E+03	4,7E-05	3,8E-05	3,0E-09	2,4E-09
	**RBD SARS-Cov-2**	8	3,5E+05	2,0E+04	1,5E-05	9,3E-06	4,4E-11	2,7E-11
	**RBD N501Y (Alpha + Beta)**	8	2,7E+05	6,2E+04	8,2E-05	4,0E-05	3,6E-10	1,9E-10
	**RBD L452R, E484Q (Delta)**	8	4,0E+05	4,6E+04	2,5E-05	4,0E-05	5,9E-11	9,4E-11
	**RBD K417N, L452R, T478K (Delta variant)**	8	4,3E+05	3,2E+04	4,0E-05	4,4E-05	9,3E-11	1,0E-10
	**RBD B.1.1.529/Omicron**	8	1,6E+05	2,6E+04	9,2E-05	7,8E-05	5,6E-10	4,8E-10
	**RBD SARS-Cov-1**	8	1,7E+05	1,4E+04	1,6E-04	9,4E-05	9,1E-10	5,5E-10
31283	**Trimer SARS-CoV-2***	8	7,4E+04	3,4E+03	2,2E-05	1,5E-05	2,9E-10	2,0E-10
	**RBD SARS-Cov-2**	8	4,0E+05	2,0E+04	8,6E-05	2,9E-05	2,2E-10	7,3E-11
	**RBD N501Y (Alpha + Beta)**	7	2,1E+05	2,8E+04	7,2E-04	5,0E-05	3,4E-09	5,1E-10
	**RBD L452R, E484Q (Delta)**	8	2,3E+05	1,9E+04	2,1E-04	4,4E-05	9,1E-10	2,1E-10
	**RBD K417N, L452R, T478K (Delta variant)**	8	3,5E+05	1,3E+04	1,2E-03	9,6E-05	3,5E-09	3,0E-10
	**RBD B.1.1.529/Omicron**	8	n.b.	n.b.	n.b.	n.b.	n.b.	n.b.
	**RBD SARS-Cov-1**	8	n.b.	n.b.	n.b.	n.b.	n.b.	n.b.
Casivirmab	**Trimer SARS-CoV-2***	4	4,0E+05	2,2E+04	3,8E-05	1,3E-05	9,5E-11	3,2E-11
	**RBD SARS-Cov-2**	4	1,1E+06	2,4E+04	5,7E-04	8,0E-05	5,4E-10	7,6E-11

	**RBD N501Y (Alpha + Beta)**	4	9,1E+04	6,0E+03	7,7E-04	4,7E-05	8,5E-09	7,7E-10
	**RBD L452R, E484Q (Delta)**	4	3,5E+05	1,4E+04	2,5E-03	2,0E-04	7,3E-09	6,4E-10
	**RBD K417N, L452R, T478K (Delta variant)**	4	7,0E+05	1,7E+04	1,8E-03	1,5E-04	2,5E-09	2,2E-10
	**RBD B.1.1.529/Omicron**	4	n.b.	n.b.	n.b.	n.b.	n.b.	n.b.
	**RBD SARS-Cov-1**	4	n.b.	n.b.	n.b.	n.b.	n.b.	n.b.
Imdevimab	**Trimer SARS-CoV-2***	4	3,1E+05	2,2E+04	3,0E-05	1,3E-05	9,8E-11	4,5E-11
	**RBD SARS-Cov-2**	4	2,6E+05	1,8E+04	3,6E-04	3,4E-05	1,4E-09	1,6E-10
	**RBD N501Y (Alpha + Beta)**	4	4,9E+05	4,9E+04	5,5E-04	6,2E-05	1,1E-09	1,7E-10
	**RBD L452R, E484Q (Delta)**	4	4,1E+05	4,0E+04	7,0E-04	1,5E-04	1,7E-09	4,1E-10
	**RBD K417N, L452R, T478K (Delta variant)**	4	2,2E+05	4,9E+04	1,9E-03	5,1E-04	9,9E-09	3,4E-09
	**RBD B.1.1.529/Omicron**	4	n.b.	n.b.	n.b.	n.b.	n.b.	n.b.
	**RBD SARS-Cov-1**	4	n.b.	n.b.	n.b.	n.b.	n.b.	n.b.
Sotrovimab	**Trimer SARS-CoV-2***	4	2,1E+04	1,8E+03	4,4E-05	3,0E-05	2,2E-09	1,6E-09
	**RBD SARS-Cov-2**	4	8,3E+04	5,8E+03	1,0E-05	1,2E-05	1,2E-10	8,3E-12
	**RBD N501Y (Alpha + Beta)**	4	7,9E+04	6,5E+03	2,2E-04	4,0E-05	2,8E-09	5,7E-10
	**RBD L452R, E484Q (Delta)**	4	7,7E+04	5,2E+03	1,0E-04	4,3E-05	1,3E-09	5,5E-10
	**RBD K417N, L452R, T478K (Delta variant)**	4	8,5E+04	2,3E+03	3,1E-05	1,2E-05	3,6E-10	1,4E-10
	**RBD B.1.1.529/Omicron**	4	3,2E+04	8,2E+02	4,8E-04	1,0E-05	1,5E-08	4,9E-10
	**RBD SARS-Cov-1**	4	1,1E+05	3,7E+03	7,7E-05	3,5E-05	7,1E-10	3,2E-10

S13 Table: Binding kinetic values of mAb.

Antibodies 29044, 31259, 31283, Casivirmab, Imdevimab, Sotrovimab binding trimeric SARS-CoV-2, RBD SARS-Cov-2, RBD N501Y (Alpha + Beta), RBD L452R, E484Q (Delta), RBD K417N, L452R, T478K (Delta variant), or RBD B.1.1.529/Omicron RBD SARS-Cov-1 measured by SPR. Values shown are mean of at least 3 replicates with standard deviation (SD).

*Affinity to trimeric protein variants is not monovalent but estimated avidity.
