# Supplementary material for: Discovery of neutralizing SARS-CoV-2 antibodies enriched in a unique antigen specific B cell cluster
Source: PLoS One. 2023 Sep 20;18(9):e0291131. doi: 10.1371/journal.pone.0291131 (PMC10511142; doi:10.1371/journal.pone.0291131)
Supplement: S4 Table — (PDF) [file pone.0291131.s014.pdf]

ST3

**Data collection and processing**	
Magnification	37000x
Voltage (kV)	300
Electron Exposure (e-/Å^2)	75
Defocus Range (μm)	0.9-1.6
Pixel Size (Å)	0.8465
Symmetry Imposed	C1
Particle Images (#)	93007
Map Resolution (Å)	3.64
FSC Threshold	0.143
**Model Composition**	
Nonhydrogen Atoms	26671
Protein Residues	3376
Nucleotides	0
Ligands: NAG	31
**Average B Factors (Å^2)**	
Protein	109.83
Nucleotide	NA
Ligand	144.22
**R.M.S. Deviations**	
Bond lengths (Å)	0.007
Bond angles (°)	0.995
**Validation**	
Molprobity Score	2.28
Clashscore	23.39
Rotamer outliers (%)	0.07
**Ramachandran plot (%)**	
Favored	93.59
Allowed	6.29
Outliers	0.12
**Model vs. Data**	
CC (mask)	0.54
CC (box)	0.74
CC (peaks)	0.46
CC (volume)	0.52
Mean CC for Ligands	0.54

S14 Table: Parameters and statistics for cryo-EM data collection, processing, structure refinement, and validation.
