# Supplementary material for: Discovery of neutralizing SARS-CoV-2 antibodies enriched in a unique antigen specific B cell cluster
Source: PLoS One. 2023 Sep 20;18(9):e0291131. doi: 10.1371/journal.pone.0291131 (PMC10511142; doi:10.1371/journal.pone.0291131)
Supplement: Attachment — Submitted filename: Rebuttal_Letter_PLOSOne.pdf [file pone.0291131.s015.pdf]

<u>Rebuttal letter</u> for the manuscript by <u>Lende</u> <u>SF</u> *et al*: "Discovery of neutralizing SARS-CoV-2 antibodies enriched in a unique antigen specific B cell cluster"

We have compiled all reviewer comments and provided each comment with an ascending number. I.e. comment 1 (C1), comment 2 (C2) etc. Immediately after each comment we have provided our answer A1, A2 etc with clear reference to changes in the manuscript and/or addition of experiment details and data.

<u>Reviewer #1:</u> This paper provides an overview of the author's neutralizing SARS-CoV-2 Mab discovery program. The paper uses the libra-based methods to improve the identification of neutralizing Abs against SARS-CoV-2, which could be interesting. Three Abs appear to have been selected as the best out of 455, but their properties are very poorly described. Due to the diffuse descriptions, both within the results section and in the materials and methods, it is unclear exactly what was performed and a clear detailed description of the critical results is not presented. Most interesting information is excluded and overview descriptions and figures that prevent a clear understanding of the work are used. As written, the paper is not appropriate for PP.

Assuming the MS was corrected, it is not the first study using libra-methods, they are not the first to identify B-cell clusters described, and they have not validated the B-cell cluster is useful for screening strategies. Presumably, the authors can provide more information on the B-cell screening and describe the quality of the final Abs they have identified to allow the reader to determine if their system has identified Abs "better" than other groups. The cryo-em data provides little relevance to the paper, beyond demonstrating one NAb is a class 1 NAb. Thus, reaching the bar for PP may be difficult. Below are a list of comments the authors may want to consider when they re-/submit to an appropriate journal.

<u>Reviewer #1:</u>
C1: The mouse study is based on SARS-CoV-2/Muchen-1.1-2020/929...which presumably is an original strain (e.g. WA-1 or Wuhan-1). Why not evaluate a VoC strain? The study needs to be performed with a control antibody that represents the current "best case" Ab for SARS-CoV-2.

A1: Yes, the SARS-CoV-2/Muchen-1.1-2020/929 is the original Wuhan-1 strain. We agree that evaluation of VoC strain(s) would have strengthen the data, however, given the continued evolution of SARS-CoV-2 we chose to use only the original Wuhan-1 strain for *in vivo* analyses and focus on emerging VoCs in the *in vitro* analyses as they were possible to add in with the pace they emerged. Regarding a control antibody, we did indeed perform a study in the K18 ACE2 transgenic mouse model using the Regeneron antibody cocktail (Casivirimab and Imdevimab) and showed that these antibodies block transmission at 40 ug/mouse (similar to the data shown for antibodies 29044 and 31283).

<u>Reviewer #1:</u> Despite the heading, I do not consider these "minor" issues.
C2: The flow experiments are not well described. There are four antigens. Cells had to simultaneously bind all 4 antigens? 4 experiments with different antigens? There are 15 samples, 5 groups? How was this completed? It is not clear from the methods either. There is no linkage of

final Abs to the patient groups and antigens that were used.

A2: We are sorry for this frustration and have tried to add description on the antigens (can be retrieved in the Answer 3 below) and the methodology in the manuscript, line 357-365.
"PBMCs from convalescent Covid-19 patients were pre-enriched for B-cells using EasySep human Pan-B-cell enrichment kit (19554, Stemcell Technologies, Cambridge, UK). B-cells were sorted by FACS on an Aria III cell sorter by sorting antigen specific memory B cells using a pool of 4 antigens as described in the Recombinant SARS CoV-2 proteins paragraph. The antigens were bound to dextramers (dCODE Klickmer, Immudex, Virum, Denmark) barcoded with a DNA oligo and coupled with both PE fluorochrome and Streptavidin. Each of the 4 antigens were mixed separately with a different DNA oligo barcode biotin-dextramer. A mix of human surface markers CD19, IgG and IgA and a live/dead cell marker were mixed with cells and 4 klickmer/antigens. Cells expressing CD19, IgG and IgA and binding to dextramer encoded antigens were sorted into bulk for 10x Genomics workflow and used for single cell sequencing with Chromium Next GEM Single Cell 5' Kit v2 (10x Genomics, CA, USA). Gating strategy is shown in supplementary Figure 1."

Further, we have compiled a large table with the description of all the selected functional verified antibodies and their group origins etc. This data has been included as supplementary data Table 1.

C3: The sequences of all protein antigens should be included.

A3: This has been added. The SARS CoV2 Wuhan strain (Wu H1) spike protein sequence derived from PDB 6VSB (Wrapp D. et. al. Science 2020, PMID: 32075877) was used for the antigen design. To make RBD proteins an Octa HIS tag -HHHHHHHH- was added for purification and an Avitag – GLNDIFEAQKIEWHE- for site specific biotinylation. For making trimerized constructs the furin site was mutated and added a GS-T4 trimerization domain – GSGYIPEAPRDGQAYVRKDGEWVLLSTFLG- as described by (Walls A.C. et. al. Cell 2020, PMID: 32155444). Further an additional Octa HIS tag -HHHHHHHH- was added for purification and an Avitag – GLNDIFEAQKIEWHE- for site specific biotinylation. The SARS1 RBD sequence was derived from UniProt accession P59596. The final protein sequences have been included in supplementary data Figure 1.

C4: What was the impact of down selection on germline sequences? IGHV3-66 and IGHV3-53 C1 Nabs are known to have limited SHM. Were they excluded during downslection? All germline seqs. should be provided and then explain how your cutoffs impacted different germ line sequences.

A4: All mAbs with somatic hyper mutation (SHM) rate lower than 2% were excluded during selection, because we focused on only somatically mutated nAbs hypothesizing that these would have higher affinity to the antigen. Due to the large number of cells to choose from, we focused on general criteria that could decrease the number of eligible mAbs (>2% SHM, only IgA or IgG1, no sequence liabilities). The germline sequences utilized in the B cell population are summarized per cell stratified by isotype in Supplementary Figure 3A, and germlines for the top neutralizing antibodies together with their rate of SHM are now supplied in Supplementary Table 1. Furthermore, how different germlines were affected by deselecting sequence liabilities is

summarized in Supplementary Figure 4. As seen, all germlines were represented uniformly compared to their abundance.
Three neutralizing mAbs from single-cell sequencing (31283, 31307 and 31331) and the mAb from previous optimization experiments, 29044, had germline IGHV3-66, thus including two mAb tested *in vivo.* 31283 had an overall SHM rate of 2.4%, thus fulfilling the selection criteria we had established. Interestingly, 31283 was in the same epitope bin as 31331, 31307, Casivirmab (utilizing IGHV3-11*01) and 29044, for which we mapped the Fab binding on the Omicron BA.1. antigen. Our other *in vivo* neutralizing antibody, 31259, had germline IGHV4-4 and was found in a different epitope bin. Sotrovimab has germline IGHV1-18*01, which is also utilized by 31897, but where sotrovimab competes only with Imdevimab and 31893, our antibody 31897 is found to bind in the same bin as 31283 and 29044. Imdevimab has germline IGHV3-33*03, shared with 31367 only, which was not included in the epitope binning experiment.

C5: p10 ,136-137 "many of the neutralizing antibodies found were sourced from cluster 8 (Fig 3B)" Aren't the statistics biased to cluster 8 because your selection was biased?

A5: We used the cluster GEX data to make conscious informed choices in the selection of monoclonal antibodies. The numbers indicated in the manuscript and in Figure 3C specifically is a simple depiction of hit-rate (i.e. how many of the antibodies selected for production from a given cluster). In this depiction the denominator is the number of antibodies from a given cluster and hence normalized to how many antibodies we selected from cluster 8 and 6 etc.

C6: P10, 157 "In comparison, the majority of mAbs (58%) display a reduction of up to 1-fold". Isn't 1-fold no change?

A6: Absolutely correct. There was a 0 missing it should read "*… up to 10-fold*". This has been corrected.

C7: P12,198 "Verification of pseudovirus neutralization was performed with full length SARS-CoV-2" I don't know what full length SARS-CoV-2 is? If authentic virus/s were used in this study, their origin BEI, specific lab, etc. should be referenced.

A7: We are sorry for this misunderstanding and have replaced "full length" with "authentic" and have included the designation.
The strain is referenced in the methods section "Neutralization assay" line 519.

C8: As the authors mention, Barnes et al have defined standard classes for Abs, which are C1, C2 etc. The use of a distinct binning nomenclature is confusing. Please exclusively use the c1, c2,c3 etc. nomenclature.

A8: Thanks this is a great point that we should have done in the first place. The standard antibody classes nomenclature defined by Barnes *et al*. has been implemented throughout the manuscript.

C9: Methods section suggest the group expressed post-fusion S protein trimers without any proline residues? As I mentioned earlier, please include the full sequences of your antigens with tags and assembly domains in a figure in the supplemental.

A9: Please see answer A3 on a similar issue. All sequences have been provided in the supplementary data Figure 1.

C10: Binding constants for SPR should be provide for the Abs tested and place in the supplement along with the sensorgrams.

A10: The binding constants for the SPR were included in the supplementary data Figure 3 (now Figure 6) but we have compiled all the additional data Kon, Koff, and KD and sensorgrams for the three most interesting mAbs and Sotrovimab which were further characterized in the *in vivo* studies. The additional binding constant data is supplied in supplementary Table 2 and sensorgrams in supplementary Figure 10.

C11: There are no data validating the modeled cryo-em structure. No FSC curve, no local resolution curve, no correlation coefficients.

A11: All the parameters and statistics for cryo-EM data collection, processing, structure refinement, and validation including correlation coefficients have been provided in supplementary Table 3.
FSC curves for refinement for density maps have also been added in supplementary Figure 11.

C12: Please provide a clear table with NT50 values for all of the "top" MAbs against all of the VoC tested.

A12: We have compiled the NT50 data for the three selected monoclonal antibodies to both SARS-CoV-1, Wuhan-B and five VoC which have been added as supplementary data Table 4.

C13: Please clearly provide the data and tables to demonstrate the binding properties and epitope

class of each of these top Abs.

A13: We have shown RBD binding (Figure 4A), VoC binding (Figure 4B), VoC neutralization (Figure 6D-F), ACE-2 competition (in Figure 4C and Figure 6A+B), SARS-CoV-1 neutralization (Figure 6D-F), and SPR competition binning data (Figure 5A+B and Supplementary data Figure 7).

Reviewer #2: In the study 'Discovery of therapeutic broad neutralizing SARS-CoV-2 antibodies highly enriched in a unique antigen specific B cell cluster' Lende et al performed a antigen-specific single cell B cell analysis from SARS-CoV-2 convalescent participants and identified a variety of SARS-CoV-2 neutralizing antibody producing B cell clusters. Then the authors cloned out several monoclonal antibodies and carried out epitope mapping, cryo-EM based structural analysis and in-vitro neutralization assay. Two potent antibodies that showed broad neutralizing ability against SARS-CoV-1 and several major SARS-CoV-2 VOC were identified and assessed in in-vivo protection against SARS-CoV-2. The authors also determined an activated memory B cell transcriptomic cluster that produced these two neutralizing antibodies. Overall, the study is relevant and could be useful for development of neutralizing mAb based broad SARS-CoV-2 therapeutics, however, there are several points in the manuscript that should be addressed.

Reviewer #2:
C14 How the B cells were sorted? If the cells were sorted for CD19, IgG and IgA and Ag-binding cells, why was a naive population present there? An example plot of the gating strategy should be included as supplemental material.

A14: We are sorry for this lack of clarity. We have provided an example of the gating strategy and included this in the supplementary data Figure 2.
To address the other question a naïve fraction present in the sorted population might be due to the size and form of the different gates generated and the stringency of the physical sorting we made using bulk and purity mode sorting. A risk of sorting cell aggregates is present although a single cell discrimination gate was pre-set. Additionally, one cannot exclude unspecific binding of some compromised cells to other cells or unspecific ligation of dextramers. Lastly, the exact phenotype of some of the sorted cells might be difficult to identify correctly based on transcriptomic profiles as the discrete features of 3-colour flow definitions become much more granular with GEX data.

C15: Overall, there is limited cataloging of the cells that encoded the neutralizing Abs. This makes it difficult to assess the benefit of using cluster identity for identifying potent and broad Abs. How many different patients did the 7176 cells come from, what was the # of cells contributed by each patient to the 7176 total? For each of the neutralizing mAbs in Figure 4, what was the associated cluster/patient? For each cell that gave rise to a neutralizing mAb, was its individual Ag score profile from the Libra-Seq (e.g. SARS-CoV-2 RBD+, SARS-CoV-1 RBD-). A supplemental table with these details for each cell or at least each of the cells that was used for the 455 mAb generation would be helpful.

A15. We are sorry for the misunderstanding. Due to Danish interpretation of GDPR rules we were not granted permission to investigate individual patients as the analysis involved GEX data and we did not have consent to perform personalized genomics data. Instead, we had to pool cells from five donors and run the analysis on three batches each including cells from 5 donors. Therefore, we are unable to track one single cell to one individual. Out of the 7176 cells that went into the selection and analysis 61,6% were from group 1, 9,1% were from group 2, and the remaining 29.3% were from group 3. All the top neutralizing antibodies were from group 1. Additionally, we have compiled data into supplementary Table 1 describing the gene usage and antigen score as requested.

C16: Detail of the methodology of how the dextramers were used for the Ag-specific B cell isolation, including dextramer, PE, and streptavidin coupling should be added.

A16: We have added more detail to the methodology in the (see also **A2**)
"PBMCs from convalescent Covid-19 patients were pre-enriched for B-cells using EasySep human Pan-B-cell enrichment kit (19554, Stemcell Technologies, Cambridge, UK). B-cells were sorted by FACS on an Aria III cell sorter by sorting antigen specific memory B cells using a pool of 4 antigens as described in the Recombinant SARS CoV-2 proteins paragraph. The antigens were bound to dextramers (dCODE Klickmer, Immudex, Virum, Denmark) barcoded with a DNA oligo and coupled with both PE fluorochrome and Streptavidin. Each of the 4 antigens were mixed separately with a different DNA oligo barcode dextramer. A mix of human surface markers CD19, IgG and IgA and a live/dead cell marker were mixed with cells and 4 klickmer/antigens. Cells expressing CD19, IgG and IgA and binding to dextramer encoded antigens were sorted into bulk for 10x Genomics workflow and used for single cell sequencing with Chromium Next GEM Single Cell 5' Kit v2 (10x Genomics, CA, USA)."

We have furthermore included the gating strategy in the supplementary data Figure 2.

C17: The process for determining Ag score is not clearly described, e.g. how well does the LIBRA-Seq – based Ag score correlate with the binding profile of the resulting mAb as presented in Figure 4?

A17: We are sorry for this lack of clarity. The Ag scores are based on the number of unique UMIs associated with the four barcode sequences that are detected from the feature barcode 10X Genomics library. The process for determining Ag score is described in the end of "Repertoire sequencing analysis" section (line 417-420):
"The antigen score (from feature barcodes) for each cell was calculated by log2p1 normalization of the number of features barcode-associated UMIs per cell, divided by the total number of UMIs for the cell, multiplied by 10,000. This is analog to the normalization performed for the GEX data but using log2 instead of the natural logarithm."

The Ag score is a continuous value which is constructed to predict the binary outcome neutralization or binding. As it is based on the number of dextramers-molecules bound to individual cells, it is a proxy for BCR binding and Ag-specificity per cell but can nevertheless suffer from unspecific binding resulting in false positives. Due to the continuous nature of the score, it is dependent on a threshold

value that discriminates the positive vs negative outcome. We thus selected receive-operator characteristics (ROC) curve to demonstrate the correlation between the score and outcome under varying thresholds. The area-under-the-curve (AUC) value represents the trade-off between false positive rate and true positive rate, where a perfect score would yield a value of 1 and a random score a value of 0.5. In Figure 3D, we show the correlation between all four scores and neutralization within cluster 8. Here we show that the SARS2 RBD score and SARS2 D614G trimer score correlated significantly with neutralization status with AUC of 0.79 and 0.7. The p-value is based on a Kruskal-Wallis test and is below 0.05 for these two (lines 136-144).

The distribution of all Ag scores is visualized in Supplementary Figure 3B (RBD Ag score) and C (SARS2 Ag score) stratified by transcriptomic cluster. These scores were the only scores that correlated significantly with predicting binding. In the main text, we describe the enrichment of Ag scores derived from RBD and SARS2 D614G in cluster 6 using the Kruskal-Wallis non-parametric test (line 116-121). Finally, the predictive power of all the Ag scores towards binding to RBD is shown in supplementary Figure 3E.

C18: Did the exclusion of mAbs to be made based on “common drug development liability sequence motifs” / CLC Main Workbench differentially impact the clusters? In other words, was there a higher rate of cells being excluded from mAb generation from one cluster vs another cluster? Also, a reference/source for CLC Main Workbench should be included in the Methods.

A18: This is a good point. We have included additional data to address this point in supplementary data Figures 4 and 5. And highlighted this in the methods section (line 415-416). Only clusters 6 and 8 were exhausted completely of liability-free unique antibodies. For these two clusters, 219 (31.74%) and 208 (33.77%) cells, respectively, were removed due to sequence liabilities. 264 cells (38.26%) and 199 cells (32.31%) had mutation rate lower than 2%. For the remaining clusters, the selection was overall made at random excluding all mAbs with liabilities to have a broad representation. The association between germline and sequence liability is summarized in Supplementary Figure 4 showing the fraction of cells with liabilities with the given germline. The fraction of cells with mAb sequence liabilities in each transcriptomic cluster is given in Supplementary Figure 5. In panel “Any liability” we see an overall uniform distribution of sequence liabilities (around 30%) for all clusters. Due to N-glyc sites often being associated with IGHV1-8, this germline was under-represented together with IGHV4-34, which was never represented due to the risk of cross-reactivity.

C19.Considering the title is “Discovery of therapeutic broadly neutralizing SARS-CoV-2 Abs…” the in vivo assessment is rather limited, testing against only single virus and also only in prophylactic and not as treatment after infection. This coupled with only a single live virus neutralization assay does not adequately support the manuscript’s title.

A19. This is a valid point. We have modified the title to: “*Discovery of neutralizing SARS-CoV-2 antibodies enriched in a unique antigen specific B cell cluster*”

C20 More details should be added to the methods or figure legends on the # of times the experiments were repeated, # of replicates, statistical tests etc.

A20. We have added number of experimental replicates to the legends and added the appropriate statistical analysis applied were appropriate.

C21. What is the cluster source of 29044 mAb? It seems to be a late add on that is not clear if it came from the LIBRA-Seq selection process.

A21. This is correct that 29044 is not sourced from the GEX cluster analysis. This has been described in the results section line 147-148.
"*An additional promising antibody, 29044, identified in a pilot run was added to the final pool of neutralizing mAb candidates"*

C22. In Figure 4, how the mAbs down-selected for analysis in A, B and C?

A22. We are sorry this was not clear. The selection was based primarily on neutralization potency. I.e the most potent antibodies were sequentially selected. However, candidates mAb with unique binding profiles were also explored further, not to miss less potent but unique binding antibodies.

C23. Sotrovimab, or similar control mAb should be included in neutralization results (e.g. Figure 4A, 6C) as a comparative neutralizing activity of these newly described mAbs.

A23. We have included Sotrovimab neutralization (of Wuhan-B and Omicron BA.1) data in the comparison table of the three top antibodies supplied in the supplementary data Table 4.

C24. Line 170 : The color description should clearly match with the image.

A24. This has been corrected.

C25. Line 187 : PDBePIS should be PDBePISA.

A25. Thanks for noticing. This has been corrected.

C26. Discussion Line 233+234: It is not clear where this breakdown of neutralizing mAbs and isotype is presented in Results.

A26. This information has been added to the results section "Monoclonal antibody selection and screening" line 144-145:
"*Antigen scores, somatic hypermutation and antibody isotype is listed for selected antibodies in supplementary Table 1."*

C27. Figure 1 : The NT50 values of the participants can be presented in groups according to the description in the method section.

A27. Thank you we have decided to keep the original representation to visualize the continuum of neutralizing capacity of all screened donors.

C28. Methods: Patient inclusion indicates 3 groups of patients; however it is not clear how this relates to the LIBRA-Seq results and mAbs generated and tested.

A28: This has not been clear and we have tried to correct this in the methods. Further, we have added a supplementary Table 1 to highlight the features of each monoclonal antibody, and we included a sentence on the proportion of analyzed single B cells going into the selection procedure in the results section (line 80-81).
