# Supplementary material for: Discovery of neutralizing SARS-CoV-2 antibodies enriched in a unique antigen specific B cell cluster
Source: PLoS One. 2023 Sep 20;18(9):e0291131. doi: 10.1371/journal.pone.0291131 (PMC10511142; doi:10.1371/journal.pone.0291131)
Supplement: Attachment — Submitted filename: PLOSOne-Tolstrup.pdf [file pone.0291131.s016.pdf]

The study by the Tolstrup lab describes the discovery and characterization of neutralizing SARS-CoV-2 antibodies. This is a very complete study with a clear and comprehendible experimental design protocol, convincing and complete data and conclusions supported by well controlled study. The manuscript can be published as submitted
