# Supplementary material for: Discovery of neutralizing SARS-CoV-2 antibodies enriched in a unique antigen specific B cell cluster
Source: PLoS One. 2023 Sep 20;18(9):e0291131. doi: 10.1371/journal.pone.0291131 (PMC10511142; doi:10.1371/journal.pone.0291131)
Supplement: Attachment — Submitted filename: Response to Reviewers.docx [file pone.0291131.s017.docx]

**Rebuttal letter** for the manuscript by Lende SF *et al*: ”Discovery of neutralizing SARS-CoV-2 antibodies enriched in a unique antigen specific B cell cluster”

We have compiled all reviewer comments and provided each comment with an ascending number. I.e. comment 1 (C1), comment 2 (C2) etc. Immediately after each comment we have provided our answer A1, A2 etc with clear reference to changes in the manuscript and/or addition of experiment details and data.

**Reviewer #1**: *The study by the Tolstrup lab describes the discovery and characterization of neutralizing SARS-CoV-2 antibodies. This is a very complete study with a clear and comprehendible experimental design protocol, convincing and complete data and conclusions supported by well controlled study. The manuscript can be published as submitted*

**Reviewer #2**: *The manuscript by Lende et al is comprehensive and detailed, with good observations and analysis of data obtained using several sophisticated techniques. The approach was to classify B cells from convalescent patients into transcriptomic profile groups. Single-cell sequence information was used to generate monoclonal antibodies via cloning, transfection and expression in ExpiCho-S cells. Resulting monoclonal antibodies were then screened for antigen binding, neutralisation activity against SARS-CoV-2 pseudoviruses and authentic viruses including major variants of concern. Finally, highly neutralising or broadly neutralising monoclonal antibodies were tested for in vivo protection against disease in a mouse model, validating the predictive value of the transcriptomic profiling approach.
The manuscript is generally well written, in clear standard English but will need thorough proof-reading to correct e.g. spacing errors.*

**Requested clarifications:**
**C 1**: *In the abstract and throughout the main body of the text, the step from characterising B cells to screening mAbs is skipped over. Only in the methods section is the generation of mAbs from the sequence data even mentioned. It would greatly assist readers’ understanding the concept of the approach used if the mAb generation was made clearer when describing the study.*

**A1**: We have added a sentence to the Result section following selection criteria of monoclonal antibodies line 131-132: “*The selection of 455 mAbs that were produced as individual antibodies is summarized in Supplementary Fig 1D.”*

**C2**: *Detailed characterisation was carried out on antibody 29044, but this was ‘identified in a pilot run’ (line 150). Were transcriptomic profiling and screening carried out on this pilot run? If they were, can the information be compiled into that for the main run? If they were not, then including this antibody as an exemplar of the profiling approach as an effective pre-screening method is inappropriate and misleading.*

**A2**: As stated the antibody 29044 was indeed selected from a smaller scale pilot run and not from the main study data. We have not tried to hide this in the description and have not assigned this antibody to any of the transcriptional clusters suggesting any linkage between the antibody 29044 and the transcriptomic selection criteria.

**C3**: *Have the authors considered population or demographic differences between studies as potentially contributing to the differences identified in lines 91-94?*

**A3**: We thank for this insightfull comment. We have not taken donor demographics into account because we were unable to perform individual (ie from a single donor) B cell sequencing due to GDPR data protection issues. Thus, we had to pool donors into groups of 5 to mask personal identifiable information. Therefore, we are unable in the current dataset to link any specific individual donor trait to a single isolated monoclonal antibody.

**C4**: *Why were switched isotypes IgG2, IgG3 and IgG4 excluded (line 125), as these were highly mutated from the germline?*

**A4**: We decided to focus on the isotypes well-represented in our data, which were IgG1, IgA1 and IgA2. This was done to focus the selection from ~8000 to 455 antibodies. As expression was only performed in IgG1-format, we aimed to represent the majority of the B cell population while simultaneously reducing the impact of changing the isotype on antibody expression. Only a small proportion of the total B cell population had IgG2 (184), IgG3 (223) and IgG4 (10) isotype (Figure 2B). While IgG4 may appear more mutated, this difference may be spurious. We specifically excluded IgG2 as it has previously been reported that IgG2-expressing B cells may respond poorly to viral antigens, and that viral responses are typically dominated by IgG1 and IgG3 (Cao *et al*., Cell Reports 2020; Irani *et al*., Molecular Immunology 2015).

**C5**: *Line 154: “The vast majority of neutralizing mAbs were RBD binders” however Figure 4A shows that most neutralising mAbs have weaker binding to the RBD than to the overall Spike.*

**A5**: Thanks for this comment. The spike antigen also included RBD and hence if there is binding to RBD there should in most cases be binding to Spike as well. There could also be small differences in immobilized antigen conformation RBD vs Spike in the recombinant well format. Importantly, Figure 4A is displayed as a heatmap. In the heatmap the binding to RBD is scaled according to the mAbs relative binding to the RBD, while spike is scaled according to Spike relative binding. Therefore the scales between antigens can not be directly compared.

We are also confident by the RBD binder statement in that almost exclusively all mAbs compete well with ACE2 for RBD binding.

**C6**: *Please include results from the in vivo dose finding studies*.

**A6**: The dose data was requested by the last round of reviewers and is included in the supplementary Figure 8

**C7**: *Line 316: So what timepoint of blood sampling is recommended for mAb discovery? Was there any attempt to correlate neutralising antibody identification with time of PBMC sampling? Table 1 describes the time from diagnosis to inclusion ie PBMC sampling, but would time from symptom onset be a better indicator for participant groups 2 & 3?*

**A7**: Thanks for this comment. As mentioned in the methods section (and as highlighted in A3) we were unable to perform individual (ie from a single donor) B cell sequencing due to GDPR data protection issues. Thus, we had to pool donors into groups of 5 to mask personal identifiable information. Therefore, we are unable in the current dataset to link any specific individual donor trait to a single isolated monoclonal antibody.

As for the symptoms onset question. In this data set there were generally very few days from symptoms onset to diagnosis as these convalescent individuals were identified very early in the pandemic when contact tracing upon exposure was very rigorous in Denmark.

**Minor comments**
**C8**: *Line 95: needs to refer to Fig 2E as well as 2D.*

**A8**: The figure reference to Fig 2E has been added.

**C9**: *Line 111: CD80 is not shown in Fig 2*

**A9**: CD80 has been deleted from the sentence.

**C10**: *Lines 162-164: add comment on BA.1*

**A10**: We have added a comment to include Omicron in this sentence (line 164-165): “…*and also generally an even greater loss of ACE2 competition to Omicron BA.1*”.

**C11**: *Line 172: There is no blue in Fig 5A. The legend and Fig 5A describe Groups A-C, and text describes Groups 1-4. Please make text consistent with figure.*

**A11**: We thank the reviewer for identifying this clitch. We have corrected the legend to Figure 5 to include the correct class descritions to mathch the text in the manuscript. Line 863- 865: “*A cut-off of 3.5 (supplementary figure 3B) was used to define epitope communities. Community class III (Greens) and class II (Oranges) compete with community class I (magenta) but not with each other.”*

**C12**: *Line 205: Gamma is not included in Fig 6D-F.*

**A12**: This is correct. We have of course deleted Gamma from the text

**C13**: *Line 206: Fig 6D-F shows BA.2, not BA.5*

**A13**: Correct. BA.5 has been corrected to BA.2

**C14**: *Line 314: “mAb” in both instances on this line is probably actually referring to “antibodies”, not monoclonals.*

**A14**: Thanks for pointing to this mistake. This has been corrected to antibodies.

**C15**: *Resolution & image quality of figures, especially Fig 2, could be better.*

**A15**: We have uploaded figures at the requested format and the requested resolution following production in Adobe Illustrator. We believe the resolution issue may be related to PDF file formation of the editorial manager system. We are however of course hapy to correct this should there be an issue.
